# The Diabetes Complications in Senior Adults (DCSA) Study: A Scoping Review and a National Case Analysis from Northern Ireland, UK on Ocular Complications in Adults Aged 80 and over Living with Diabetes

**DOI:** 10.1007/s44402-026-00197-y

**Published:** 2026-09-29

**Authors:** Laura N. Cushley, Katie Curran, Basil Saju, Josh Kamalarajah, Max Kamalarajah, Alan Sproule, Soma Lengyel, Tunde Peto

**Affiliations:** 1 Centre for Public Health, Queen’s University Belfast, Belfast, UK; 2 School of Medicine, Dentistry and Biomedical Sciences, Queen’s University Belfast, Belfast, UK; 3 Peninsula Medical School, University of Plymouth, Plymouth, UK; 4 Department of Ophthalmology, Belfast Health and Social Care Trust, Belfast, UK

**Keywords:** Diabetes, Diabetic retinal disease, Elderly, Over 80s

## Abstract

**Background:**

The number of people 80 years and over with a diagnosis of diabetes is increasing. This is due to people with type 1 diabetes living longer, successful diabetes management and a growing aging population. Due to this increase, there is limited understanding of the impact of diabetes in people 80 years or over, especially in relation to diabetic retinal disease (DRD) and other ocular complications.

**Methodology:**

A scoping review was conducted using PRISMA-ScR guidelines, with two reviewers independently screening abstracts and full texts. Case study data were obtained from the Northern Ireland Diabetic Eye Screening Programme.

**Results and Summary:**

Database searches identified 1644 references, of which seven studies met the inclusion criteria following review. Across these, 29,734 participants were included, with 14,920 (50%) aged 80 years and over. The reported prevalence of diabetic retinopathy (DR) and diabetic maculopathy (DM) varied considerably across studies, ranging from 0.8% in Australia to 9.5% and 11.6% in Thailand and Suriname, respectively. This substantial variation may be attributable to differences in participant characteristics, study populations, geographical and regional settings, diagnostic and grading criteria for DR and DM and other methodological factors.

Some studies reported the incidence of DRD in people 80 years and over was higher in those with a longer duration of diabetes. However, none have provided a comprehensive understanding of the burden of both diabetes-related and other ocular diseases within this older patient cohort. This case study found that approximately 20% of individuals aged 80 years and over had a diagnosis of diabetes. Although the proportion with proliferative, sight-threatening diabetic retinopathy was small (0.6%), this rate is comparable to the general population of people with diabetes.

**Conclusion:**

Few studies have examined DRD in people 80 years and over. This case study aligns with the limited available evidence and highlights the continued importance of screening in this population.

Key Points

- There is limited knowledge on eye complications, specifically diabetic retinopathy and maculopathy, in people with diabetes aged 80 years and over.
- Current evidence shows a large variation in levels of diabetic retinopathy and maculopathy between studies from different contexts and countries, from 0.8% to 11.6%.
- A Northern Ireland case study showed that 20% of the 80 years and over population had diabetes, with 0.6% having sight-threatening diabetic retinopathy, similar to the percentage in the general diabetes population.

## Background and Current Literature

The diabetes landscape is constantly changing, with the number of people affected by diabetes increasing worldwide. People with diabetes, just like the general population, are living longer, albeit with more chronic disease burden, complications and comorbidities. The discovery of insulin over 100 years ago and the subsequent options of diabetes medications have been a game-changer for diabetes care and enabled not only longer life for most, but better quality of life as well for some.

Diabetes mellitus still remains a major global concern [1], affecting over 589 million adults, predominantly with type 2 diabetes (T2D) and including about 9.5 million people with Type 1 diabetes (T1D) [2]. Individuals with diabetes are at risk of various chronic neurovascular complications, including diabetic retinal disease (DRD), mainly Diabetic Retinopathy (DR) and Diabetic Maculopathy (DM).

The International Diabetes Federation (IDF) suggests that 11.1% of adults (aged 20–79 years) are living with diabetes. However, there is limited information on those outside of this age bracket, i.e., aged 80 years and over. It is estimated that 1 in 5 people over 65 years of age will have a diagnosis of T2D, contributed to by genetics, lifestyle, increasing obesity and improved survival of those with chronic diseases such as diabetes [3].

The true prevalence of diabetes is lacking in those over 80 years of age, given that they are often excluded from large cross-sectional studies and controlled trials due to their age, complex comorbidities and frailty [4–6]. Elderly people, especially those 80 years and older, often have other comorbidities, including mobility problems, hearing loss and cognitive impairment [5–7], which can all hinder access to healthcare facilities, including diabetes retinopathy or maculopathy screening.

With regard to DRD, globally DR-related blindness has increased significantly over the last three decades in the general population, and is estimated to quadruple by the year 2050 in people over 65 years of age [8, 9]. While DR screening has helped to reduce the number of people going blind due to DRD globally [10], research suggests that older people often find attending screening appointments difficult, which in turn can cause delays in diagnosis and treatment and raise the risk of visual loss [7, 11–14].

This paper includes the first scoping review of currently available DRD literature on people aged 80 years and over with diabetes and their ocular complications. The findings of this review are strengthened by adding original research of a nationwide Northern Ireland case study of individuals ≥80 years old with diabetes, exploring their ocular complications and levels of DR and DM.

## Review Methods

A scoping review methodology following the Preferred Reporting Items for Systematic Reviews and Meta-Analyses (PRISMA) guidelines [15]. Additionally, the PRISMA extension for scoping reviews (ScR) Checklist was used [16] for the scoping review (Supplementary File 2). The PCC Framework was used: Population (people 80 years and over with a diagnosis of diabetes), Concept (DRD levels—including DR and DM) and the Context (globally) [17]. An information specialist (RF) was consulted to ascertain the keywords, Boolean operators and databases for the search informed by the PCC above. The study protocol was registered on the Open Science Framework for transparency.

Medline ALL, Embase and Web of Science were searched on 6th December 2024 using the search terms outlined in Supplementary Table 1. Database searches conducted resulted in 759 papers from Embase, 759 from Medline All and 126 from Web of Science. All references were downloaded using a RIS file and uploaded into Covidence software (covidence.org) for screening. All studies were screened independently by two reviewers (BS and LC) using inclusion/exclusion criteria outlined in Table 1 below, along with their justification:

**Table 1:** Inclusion and exclusion criteria.

Inclusion criteria	Exclusion criteria
• Adults aged 80 years and over (as this population is classified as the elderly who face unique ocular complications associated with diabetes in which research/data is limited). • Studies where diabetes is a primary concept and not simply a reported variable. • Studies where eye complications are a primary concept alongside the diabetes. • Original research (these include studies such as cohort/observational studies and ensure that the findings are based on primary sources of data collection rather than secondary summaries).	• Exclude those under 80 years of age (studies with a younger population with diabetes are excluded as they may not reflect the same patterns in ocular complications as seen in those aged ≥80 years. • Exclude non-English studies (these are excluded to ensure accurate interpretation and accessibility). • Any animal studies (this review only focuses on human populations). • Post-mortem studies (this does not contribute to understanding of ocular complications in living individuals which this review is based on). • Studies where diabetes is only an additional collected variable (excluded to maintain specificity of the review).

All conflicts were discussed by both reviewers, and where a conflict could not be resolved, a third reviewer (KC) was consulted. Full-text screening was conducted by two reviewers (LC and BS) and conflicts were resolved in the same way.

Data extraction was conducted (by BS and LC) into a Microsoft Excel file (Microsoft.com). Due to the scarcity of studies specifically related to diabetes and ocular complications in elderly people with diabetes, there were no studies where all data could be extracted. In addition, from the population-based studies or those on visual impairment and blindness registers, it was difficult to extract information on other ocular complications in people with diabetes aged ≥80 years. Therefore, LC and BS extracted data on levels of DR and DM in the relevant age group. The data variables extracted were author, publication year, study title, study design and country, total study sample size, sample size ≥80 years old and DRD rates.

## Review Results

**Fig. 1:**
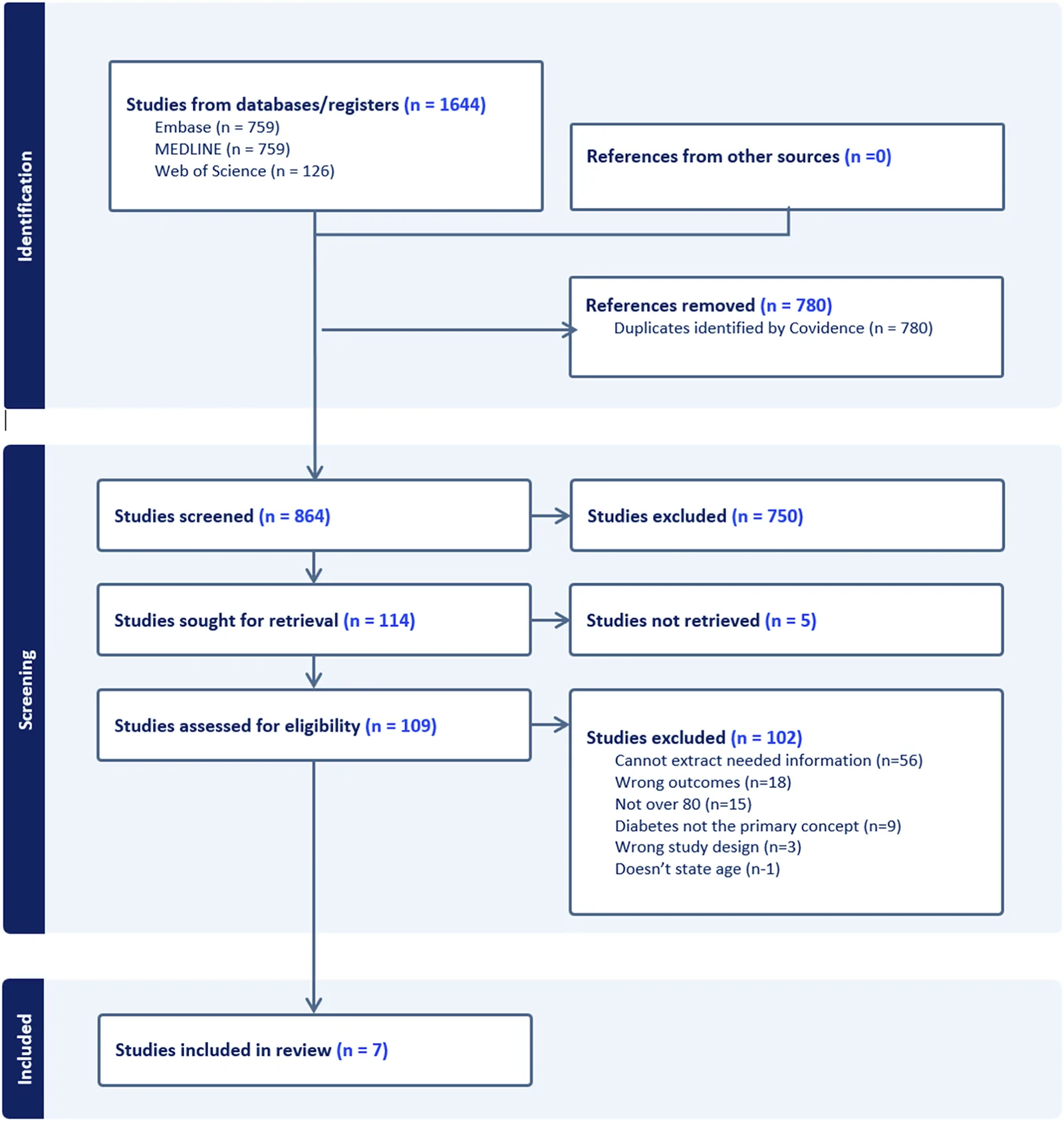
Preferred Reporting Items for Systematic reviews and Meta-Analyses (PRISMA) Diagram for Extracted Studies.

A total of 1644 references were uploaded into the Covidence platform, where 780 duplicates were identified and removed automatically by the system. A total of 864 studies were title and abstract screened, with 750 removed due to not meeting the inclusion criteria. A total of 109 papers were retrieved and assessed for eligibility, with 102 studies excluded due to the population in the study not being ≥80 years (15), wrong outcomes (18), age not defined (1), wrong study design (3) or diabetes not the primary concept being studied (9). Most were excluded as data could not be extracted for only those aged ≥80 years (56) as per the PRISMA diagram (Fig. 1). A total of seven studies were included in the review.

The seven studies were published between 2000 and 2016, with two (28.6%) conducted on a diabetes specific population and the other five (71.4%) centred around visual impairment and blindness. Four studies were population-based, one was a retrospective analysis, one a cohort longitudinal analysis and one was an add-on study to the Medical Research Council (MRC) trial. The geographical distribution of the studies primarily featured high-income countries, with research contributions from the United States (two studies), Europe (two studies – Germany and the United Kingdom) and Australia (one study), with one study from Thailand and one from Suriname, lower- income nations.

Across all seven studies, there were a total of 29,734 participants, of whom 14,920 (50%) were aged ≥80 years. T2D was the focus of three studies [18–20] and diabetes type was unspecified in four others [21–24]. All studies included had information regarding DRD in the ≥80-year-old population. Two studies reported on levels of visual impairment and blindness in the ‘over 80s’ due to DRD [23, 24]. Evans et al. and Finger et al. [23, 24] reported rates of visual impairment and blindness caused by DRD of 3.3% and 5.3%, respectively, in the ≥80 year age group. Evans et al. reported that DRD accounted for only 3.4% of visual impairment and blindness in the total population. Finger et al. observed that age-related macular degeneration (AMD) was the leading cause of blindness in the ≥80s, whilst glaucoma and DRD contributed less in very old individuals, and that DRD prevalence decreased with age [23].

Huang et al. [20] published a larger longitudinal study conducted in the United States, showing that DRD incidence for those ≥80 years of age was 14.9 per 1000 person-years with a diabetes duration of ≥10 years versus 7.99 per 1000 person-years in the same age group, but with a shorter diabetes duration (0–9 years) [20]. Their study suggested that DRD rates declined slightly with age; however, it is still a significant burden for those ≥80 years of age [20]. Another USA study conducted on 2000 older licensed drivers (28% of the study population was ≥80 years) showed that the DR prevalence in the ≥80s was 1.6%, and 0% in the ‘over 90s’ [22], showing a low overall prevalence rate in this highly mobile and competent population of still active participants. Similarly, an Australian study showed a 0.8% DRD prevalence rate in those ≥80s [21].

In contrast, a small study on elderly priests in Thailand showed a higher rate of sight-threatening DR, with 9.5% having no DM (two of 21 people) [19], correlating with longer diabetes duration, poor glycaemic control and hypertension [19]. In Suriname (South America), diabetes prevalence in the ≥80s was lower than in the general population (11.6% vs 24.6%) [18]; however, DR prevalence was high (30.8%), with 8% having sight-threatening DR in the older group [18].

## Review Discussion

These results confirmed the scarcity of literature on DRD prevalence and incidence, especially with regard to DM in people aged 80 years and over. It is important to note that several of these papers reported differing levels of DR and DM but have very different global contexts, including variations in life expectancy and socio-economic contexts. In addition, some of these studies report on a very specific population, for example, drivers 70 years and over in the USA [22] and priests in Thailand [19], which are very different. In addition, because senior adults are not generally included in clinical trials or large epidemiological studies [4–6], the current literature may have some biases and is based on potentially opportunistic or retrospective data collection.

The scoping review also identified only one study that investigated a longitudinal analysis of older people over 60 years with T2D and reported a higher risk of DRD in people with T2D and longer diabetes duration [20]. This study also identified that while there was a risk of DRD in older people with diabetes, it was lower than in younger people with diabetes. This is in keeping with other studies which identified low percentages of DR in people aged ≥80 years and suggested an even lower percentage in people aged ≥90 years. The most reliable data on DRD levels come from high-income countries (United States and Australia) with well-established healthcare systems. These results may reflect survivor bias and be influenced by earlier good metabolic control. In low- to middle-income countries, DR rates were higher (between 8–9% vs 0.8–1%), most likely reflecting a disproportionate burden of diabetes and the suboptimal metabolic control and complication screening profile of the countries [25]. Interestingly, reports from the USA and United Kingdom show that the percentage of ≥80-year-olds who are visually impaired or blind due to DRD is low, accounting for approximately 3–5% of those who are blind, in contrast to AMD contributing to about 80% [23, 24].

Underreporting may also be present given that national surveys in Low- and Middle-Income countries (LMICs) are potentially missing or do not focus on specific causes of visual impairment, including retinal diseases such as DR.

Some identified studies showed that DR prevalence declines with age, which could be attributed to mortality selection bias and higher mortality in people with diabetes before reaching ≥80 years [22, 23]. Until recently, it was highly unlikely for people with diabetes to live past 80 years of age [26], explaining the lack of studies of people aged ≥80 years. However, the number of people living longer is increasing, with an average life expectancy of 78.8 years for males and 82.8 years for females in the UK [27]. Many older people are being diagnosed with diabetes due to age-related issues such as reduced insulin secretion and increased insulin resistance caused by changes in body composition [28].

In high-income countries, there is a focus on AMD and glaucoma, which are the primary causes of visual impairment and blindness in those ≥80s years of age [23, 29]. Thus, the lack of attention to diabetes-related eye complications in the elderly is likely to contribute to underreporting.

Therefore, there is a need to explore this in more detail, as shown in the case study reported below.

## Case study: Ocular Complications in People ≥80 Years with a Diagnosis of Diabetes in Northern Ireland

An audit of all individuals aged ≥80 years with a diagnosis of diabetes in the Northern Ireland Diabetic Eye Screening Programme [30] (NIDESP) was conducted (Audit registration number: 7369). Data were obtained in March 2024, updated in September 2025 and included age, gender, type of diabetes, diabetes duration, year of last screening, last screening outcome, previous DR and DM grade and presence of other ocular diseases. Worse eye DR and DM grades were used for analysis. Data were analysed for frequency using IBM SPSS Statistical Package (Version 28, ibm.com). Further statistical analysis was conducted on each age cohort (80–89 and 90+) using Pearson’s chi-squared tests and odds ratios.

There were 16,770 people ≥80 years with a diagnosis of diabetes registered on the NIDESP database. With an estimated population of *85,692* aged over 80 years taken from the NISRA database [31], the prevalence of diabetes was estimated to be 19.57% in those ≥80 years of age in Northern Ireland.

Of the 16,770 people, 8082 (48.2%) were male and 8688 (51.8%) were female. The mean age was 85.78 years, median was 85 years (SD 3.7, 95% [85.69, 85.86]). The majority (92.3%) had type 2 diabetes, only 1.3% had type 1 diabetes and 0.01% had Maturity Onset Diabetes of the Young (MODY); 5.4% did not have a diabetes type specified. The mean duration of diabetes was 10.81 years, with a median of 8 years (range 1–64 years, SD 8.5 and 95% CI [10.51, 11]). Overall attendance, in all NIDESP pathways, from this age group was good with 85% of the patients attending with a carer or family member.

Previous screening grades were available for 16,062 people, and the breakdown can be seen in Table 2*below*. The NIDESP system follows the UK National Screening Committee (UK NSC) grading system using R0, R1, R2, R3A, R3S and M0, M1 – more information on this grading system can be found within the references [32, 33]. Of these, most (67.3%) had no DR or DM (R0M0 UK NSC). About a quarter (24.5%) had background diabetic retinopathy (BDR) (R1 UK NSC) with no maculopathy (M0). A very small percentage (0.6%) had active proliferative DR (R3A), with a third of those having diabetic maculopathy (M1). A small number were ungradable (3.8%) were ungradable due to media opacities.

**Table 2:** Retinopathy and maculopathy grades.

Retinopathy/Maculopathy level [33, 34]	Number	Percentage
R0M0	10,805	67.3%
R1M0	3942	24.5%
R1M1	323	2%
R2M0	96	0.6%
R2M1	87	0.5%
R3AM0	64	0.4%
R3AM1	34	0.2%
R3AM0	89	0.6%
R3AM1	12	0.1%
Ungradable	610	3.8%

Those with more advanced disease had a longer duration of diabetes, with those having either active or treated proliferative DR (R3A- active and R3S – stable) having a mean diabetes duration of 25 years (median 25 years). Of those with moderate to severe DR, the mean diabetes duration was slightly less, at 21 years (median 18 years).

A total of 946 (5.8%) people were cared for in hospital eye services, 303 of those for DRD and 643 for other, non-DR-related ocular complications. A small number, 6.5% (1734 people), had other ocular diagnoses recorded, most commonly cataract (40%) and AMD in 18.4% of the cohort.

In both age cohorts, ocular comorbidities were strongly associated with ungradable retinal images. Among individuals aged 80–89 years old, the presence of ocular co-morbidities was associated with a nearly 13-fold increase in the odds of ungradable images (odds ratio 12.9; χ²(1) = 1197.5, *p* = 0.0001). In the over-90s cohort, ocular co-morbidities remained significantly associated with ungradable images, although with a reduced effect size (odds ratio 6.2; χ²(1) = 66.6, *p* = 0.0001).

## What do People over 80 Years with Diabetes Think About Their Eyes?

A small sample of people aged ≥80 years with diabetes attending a weekend screening clinic were surveyed. This was an opportunistic audit sample within a screening clinic. The questionnaire was created by the study team and reviewed by five people with diabetes to ensure validity and accessibility. The questionnaire included demographic questions together with a Likert rating scale of knowledge of diabetes, diabetes management, DRD knowledge and screening preferences.

A total of 31 consecutive people of this age group responded: 16 females and 15 males. All respondents had T2D diabetes and the average age was 83 years (range 81–93 years); the average diabetes duration was 14 years (range 2–32 years). Most, 74% (23), managed their diabetes using tablets and a majority (80%) managed their own medication. Over half (58%) relied on the nurse in the general medical practice for ongoing diabetes care. Almost three-quarters (74%) did not know of the existence of potential diabetes eye complications before their first DR screening appointment.

Respondents self-reported an average knowledge of diabetes of 4.6/10, knowledge of management of their diabetes scored 4.7/10 and knowledge of complications scored lower at 3.6/10. Around half (45%), wanted more information on how diabetes could affect their eyes, with 92% wanting information verbally by phone or through in-person information sessions. When asked what times suited best for screening appointments, 74% stated weekends were best with the others stating either weekdays or weekends, depending on the availability of the person bringing them in.

## The Impact on Future Care

It is clear from this study that there is a high prevalence of diabetes (19.57% of the older population), with a quarter of people aged ≥80 years having some level of DRD. This further supports that screening is necessary in this population. Given the large number of media opacities in this age group, it is important that screening services have pathways to enable effective screening and slit lamp services in this cohort. In addition, the study showed that diabetes knowledge is low in this population. Therefore, better education, leading to potentially improved diabetes management and empowerment, could benefit people within this cohort.

## Concluding Discussion

These data show that nearly 20% of the population over 80 years of age have a diabetes diagnosis, significantly more than the overall population estimate of 10–14% in the UK [34]. Given the accuracy of the Northern Ireland (NI) Diabetic Eye Screening Programme database, we believe that this estimate is likely to reflect the higher prevalence in this age group. There might be a slight underestimation, but given the high level of use of health services in this age group, it is unlikely that substantial numbers of patients are undiagnosed. The present data show similar results to those found in other high-income countries such as Italy and the USA [35, 36]. Having 1:5 people in this age group living with diabetes is likely to have a major impact on the overall health of elderly people, such as an increased risk of vision loss, impaired mobility and cognitive function [37], all leading to increased healthcare worries and healthcare usage. Diabetes management becomes challenging once these complications occur, with reduced ability to read instructions, drug labels and insulin dosage, deciphering food labels and performing foot care. These can potentially have a negative impact on mental health and other health outcomes, and as most attend multiple appointments (85% attend with a carer or family member, or are brought by ambulance), there is an additional burden of care and emotional stress on the family and social services. There is also the high economic cost of diabetes, especially in the elderly, with primary and secondary healthcare costs two to three times more than the general population [38, 39].

In the case study presented here, the mean duration of diabetes was 11 years, meaning many of these people were diagnosed later in life. On average, DR complications are more likely when a patient is diagnosed after 30 years of age, with 30% having DR at T2D diagnosis and 60% after having DR for 15 years [40, 41]. Some have suggested that a majority of the elderly diabetes population will have DR of some type in their lifetime [42]. In contrast, the current study shows that around a quarter of the sample of over 80 s with diabetes had background retinopathy (R1 levels). However, only 2.3% had Sight Threatening Diabetic Retinopathy (STDR) (R2 and above), with only 0.6% having active referrable proliferative DR. The results showed that more than twice as many of the elderly were referred to hospital eye services due to other ocular diseases besides DRD. These results also showed that the mean duration of diabetes was longer in those with STDR compared to individuals with no or background DR, as shown in other investigations [20]. Much like the findings from other studies, cataract and AMD (19%) were the biggest causes of referral [23, 24]. This agrees with reports that AMD is the most common cause of visual impairment in many high-income countries, including Great Britain and Northern Ireland [29]. The presence of retinal vein occlusion may impact image grading and impair distinguishing relevant pathologies. In addition, a quarter (25%) of the patients had media opacities necessitating additional slit lamp examinations, as well as subsequent cataract surgery referral, adding to the patient’s, healthcare systems and the societal burden.

Diabetic eye screening clearly remains important within this population, not only to establish DRD levels but also to identify other relevant eye conditions. The improvement in survival rates in those aged 80 years and over with diabetes is one of the major public health achievements of the last century. It is now imperative to be equipped to look after these patients better, so that services can be tailored to their needs or new services established where necessary. This will resonate, not only with the patients and their carers, but also with the well-understood goals of Healthy Ageing in our society [43].

## Supplementary information

Supplementary Table 1_OPO

Supplementary File 2_Over80sOcular

## Data Availability

The data supporting the findings of this study are not publicly available due to ethical restrictions and the need to protect the privacy of study participants. Data may be made available from the corresponding author upon reasonable request, subject to approval by the relevant ethics committee.
