# Supplementary material for: The Diabetes Complications in Senior Adults (DCSA) Study: A Scoping Review and a National Case Analysis from Northern Ireland, UK on Ocular Complications in Adults Aged 80 and over Living with Diabetes
Source: Ophthalmic Physiol Opt. 2026 Sep 29;46(6):1289–95. doi: 10.1007/s44402-026-00197-y (PMC13694538; doi:10.1007/s44402-026-00197-y)
Supplement: Supplementary file 1 [file 44402_2026_197_MOESM1_ESM.docx]

| **Medline ALL** | **Embase** | **Web of Science** |
| --- | --- | --- |
| Total Hits - 759 | Hits - 759 | Hits - 126 |
| **Search Terms** | **Search Terms** | **Search Terms** |
| Eye Diseases/ or eye disease* OR Ophthalmic  AND  Diabet* OR diabetes mellitus OR Diabetes Mellitus, Type 2 OR  Diabetes Mellitus, Experimental  OR Diabetes Mellitus, Type 1 | eye disease OR  ophthalmic  AND  diabet* OR diabetes mellitus OR experimental diabetes mellitus OR  insulin dependent diabetes mellitus  OR non-insulin dependent diabetes  mellitus  AND  very elderly OR over 80*  OR  senior* OR elderly OR geriatric* | "eye disease*" OR ophthalmic  AND  diabet*  AND  "over 80*" OR senior* OR elderly OR geriatric* |
| **Limits** | **Limits** | **Limits** |
| limit to (english language and humans)  limit to "aged (80 and over)" | limit 10 to (human and english language) |  |

**Supplementary Table 1: Search Terms and hits for each database**
